# Lecture Capture, Transcripts, and Captioning in US Colleges of Osteopathic Medicine: Descriptive Cross-Sectional Survey

**DOI:** 10.1007/s40670-024-02224-4

**Published:** 2024-11-12

**Authors:** Phillip Khong, Duncan Holmes, Bahar Masoudian, Gregg C. Lund, Steven Garwood

**Affiliations:** 1 College of Osteopathic Medicine, Rocky Vista University, Parker, CO USA; 2 Rowan-Virtua School of Osteopathic Medicine, Rowan University, Stratford, NJ USA; 3 College of Osteopathic Medicine, Touro University, Vallejo, CA USA; 4 Park City, UT USA; 5 Rowan-Virtua School of Osteopathic Medicine, Academic Affairs, Rowan University, Stratford, NJ USA

**Keywords:** Lecture capture, Lecture recording, Transcription, Closed captions, Accessibility

## Abstract

This study describes lecture capture, transcription, and captioning services at US Colleges of Osteopathic Medicine (COMs). An anonymous online survey was sent to Deans or Directors of Curriculum at all 34 fully accredited COMs, with 21 (62%) responding. Results showed widespread lecture recording (95%), but of those, there were varied transcript and caption offerings: 7 (33%) provided both, 5 (24%) offered closed captions only, and 3 (14%) offered transcripts only. Overall, 71% offered at least one service. These findings indicate the prevalence of these lecture services at US COMs and will assist COMs in benchmarking their practices.

## Background

Lecture remains a common instructional method in medical education [1–3] and remains popular, especially among first- and second-year students [2, 3]. Lecture capture is also extremely popular for students across higher education [4–10]. Students use lecture recordings for multiple purposes, such as viewing missed lectures, studying for exams, note revision, and otherwise managing the learning process [4, 7–11]. Recent research on medical school students indicates that students often study by re-watching lectures and that approximately 30% of medical school students recommend re-watching lectures as a study strategy [2]. As lecture is a popular teaching and learning method, and students utilize lecture recordings for learning and preparing for assessments, it is vital to ensure they are as usable and accessible as possible.

One potential method to add utility and improve accessibility is to provide transcripts and captions for lecture recordings. While postsecondary institutions are required by Sect. 504 of the Rehabilitation Act of 1973 and the Americans with Disabilities Act of 1990 to offer equal access and reasonable accommodations to students with disabilities [12], providing transcripts and captions can benefit all students. CAST, formerly the Center for Applied Special Technology, is a group that focuses on Universal Design for Learning (UDL), a framework “to improve and optimize teaching and learning for all people based on scientific insights into how humans learn” [13]. UDL specifies the benefits of providing transcripts and captions. Checkpoint 1.2 in UDL, “Offer alternatives for auditory information,” suggests explicitly the addition of text equivalents, to improve the utility of auditory-focused learning materials. There is sparse research in the higher education literature on student use of transcripts and closed captions with lecture recordings [4, 7]. In a 2020 systematic review on the topic of lecture capture, transcripts and closed captions were not indicated as an area of active study [4]. Studies that have explored transcript and caption use indicate that students use transcripts to read instead of listen (to speed review), search the transcripts to efficiently find content, and utilize transcripts when the presenter speaks rapidly or whose pronunciation does not reflect their linguistic background [6, 14]. Overall, students found transcripts and captions helpful and that they afforded “efficient learning” [6].

Given the popularity of lecture capture in medical education and the potential benefits of transcripts and captions, it is important to understand the current landscape of these services in Colleges of Osteopathic Medicine (COMs).

## Activity

This descriptive cross-sectional survey study employed a brief, seven-question, anonymous survey distributed to Deans or Directors of Curriculum at the 34 fully accredited main campus COMs in the USA as of January 14, 2024. The survey was constructed, distributed, and managed using Qualtrics, a web-based survey tool. The survey focused on the availability of recorded lectures, as well as operational aspects and student training related to lecture capture, transcription, and captioning services. This study was approved by the Rowan University Institutional Review Board (IRB) under protocol number PRO-2024–129.

The survey consisted of questions addressing the following areas:

1. Frequency of lecture recording and provision to students
2. Availability of transcripts and/or closed captions
3. Methods of creating transcripts (if provided)
4. Methods of creating closed captions (if provided)
5. Review/editing process for auto-generated transcripts or captions
6. Student training on accessing and utilizing lecture recordings, transcripts, and/or closed captions
7. Additional information about lecture capture, transcript, or closed-captioning processes

The link to the survey was active for approximately 30 calendar days, with reminder emails sent to non-responders on days 7, 14, and 28. Participation was voluntary, and the survey was designed to take no longer than 2 min to complete. Participants did not receive any form of compensation for completing the survey. The recipient was informed in the recruitment email that if there was another administrator who could better complete the survey, they could forward the email to that person. Due to the anonymous method, no data was collected to measure how often the email was forwarded.

Data analysis consisted of descriptive analysis of quantitative responses and qualitative analysis of text responses. All data were reported in aggregate to protect confidentiality.

## Results

Of the 34 COMs surveyed, 21 responded (61.8%). Lectures were frequently recorded. Of responding COMs, 11 (52%) always record class lectures, 9 (43%) record lectures most of the time, and 1 (5%) often records lectures (Table 1).

**Table 1:** Survey questions and responses (*n* = 21) regarding lecture recording practices at US Colleges of Osteopathic Medicine (COMs)

Survey questions	Responses to lecture capture survey questions					
1) How often does your COM record class lectures and provide them for student use?	**Never**	**Rarely**	**Sometimes**	**Often**	**Most times**	**Always**
	0 (0%)	0 (0%)	0 (0%)	1 (5%)	9 (43%)	11 (52%)
2) Are transcripts and/or closed captions (CC) provided for the recorded lectures?	**Transcript only**		**CC only**		**Both transcripts and CC**	**Does not provide either**
	3 (14%)		5 (24%)		7 (33%)	6 (29%)
3) If transcripts are provided, how are they created? (Select all that apply)	**Auto-generated by lecture capture software**	**Manually created/edited**	**Outsourced to 3rd party transcription service**	**Other**	**Not applicable**	
	10 (83.3%)	1 (8.3%)	1 (8.3%)	0	9	
4) If closed-captions are provided, how are they created? (Select all that apply)	**Auto-generated by a lecture capture software**	**Manually created/edited**	**Outsourced to a third party transcription service**	**Other**	**Not applicable**	
	12 (92.3%)	0 (0%)	1 (7.6%)	0	8	
5) Is there a review/editing process to check the accuracy of auto-generated transcripts or captions?	**No**		**Yes**		**Not applicable**	
	14 (93.7%)		1 (6.3%)		6	
6) Are students provided training on accessing and utilizing the lecture recordings, transcripts, and/or closed captions?	**No**		**Yes**		**Not applicable**	
	3 (20%)		12 (80%)		6	

The provision of transcripts and/or closed captions varied among the institutions. Both transcripts and closed captions were provided by 7 (33%); 5 (24%) provided closed captions only; 3 (14%) provided transcripts only; and 6 (29%) did not provide either transcripts or closed captions.

For those COMs that provide transcripts, the creation methods differ. The majority (10 (83.3%)) use transcripts created by automatic speech recognition (ASR) within the lecture capture system. Manual creation/editing and outsourcing to third-party services are less common (1, 8.3% each). A similar trend is seen in the creation of closed captions, with 12 (92.3%) using ASR captions from lecture capture software. Notably, no COMs reported manually creating or editing captions, and 1 (7.6%) outsourced to a third-party service.

Only one (6.3%) of COMs providing transcripts or captions reviewed these materials prior to distribution.

Of the 15 COMs that reported providing lecture recordings and transcripts and/or captions, 12 (80%) provided training to students on accessing and utilizing these tools. This training is most commonly provided during orientation prior to the start of the first year.

Because there were limited free-text responses, we did not analyze this data.

## Discussion

The results of this study provide valuable insights into the current state of lecture capture, transcription, and captioning services in Colleges of Osteopathic Medicine. The high adoption rate (95% of responding COMs recording lectures always or most of the time) aligns with the popularity of lecture capture among medical students [2, 10].

However, there is significant variation in the provision of transcripts and closed captions. While 71% of responding COMs offer some form of transcript or caption, nearly a third (29%) do not provide either. This presents an opportunity for improvement, especially considering the potential benefits of transcription and captioning to make recordings more usable and accessible.

The heavy reliance on automatic speech recognition (ASR) for transcripts and captions (83.3% for transcripts and 92.3% for captions) raises questions about accuracy, particularly given that only 6.3% of COMs reported having a review/editing process in place. While constantly improving, ASR technology faces accuracy challenges with scientific jargon, speaker accents, and rate of speech [15]. This lack of accuracy can result in errors, potentially impacting the effectiveness of these tools for student learning.

It is encouraging that 80% of responding COMs provide training to students on accessing and utilizing lecture recordings, transcripts, and/or closed captions. However, there is room for improvement, as nearly a fifth of COMs do not offer adequate training.

The limitations of the study include the response rate of 62%, potentially not understanding the entire COM environment. Additionally, the survey did not explore in-depth aspects, such as the specific platforms used, the quality of the transcripts/captions, or the effectiveness of student training programs. Finally, the addition of medical schools granting the MD degree would offer a more complete assessment of the medical educational settings in the USA.

Future research could focus on:

1. Assessing the impact of providing transcripts and captions on student learning — including students with disabilities
2. Exploring best practices for integrating lecture capture, transcription, and captioning services into curricula and student study practices
3. Evaluating the accuracy of ASR transcripts and captions

This study provides a snapshot of the current landscape of provision, operations, and training of lecture capture, transcription, and captioning services in Colleges of Osteopathic Medicine, a significant site for training US physicians. While there is widespread adoption of lecture recording, there are opportunities for improvement in the provision and quality control of transcripts and captions. As these tools continue to play an important role in medical education, further research and development in this area could significantly enhance the learning experience for osteopathic medical students.
